# Pharmaceutical studies on and clinical application of olanzapine suppositories prepared as a hospital preparation

**DOI:** 10.1186/s40780-016-0055-6

**Published:** 2016-09-21

**Authors:** Kazuaki Matsumoto, Satoru Kimura, Kenichi Takahashi, Yuta Yokoyama, Masayuki Miyazawa, Satoko Kushibiki, Morio Katamachi, Junko Kizu

**Affiliations:** 1 Division of Practical Pharmacy, Keio University Faculty of Pharmacy, 1-5-30 Shibakoen, Minato-ku, Tokyo, 105-8512 Japan; 2 Department of Pharmacy, Shonan Central Hospital, 1-3-43 Hatori, Fujisawa-shi, Kanagawa 251-0056 Japan; 3 Department of Palliative Care, Shonan Central Hospital, 1-3-43 Hatori, Fujisawa-shi, Kanagawa 251-0056 Japan

**Keywords:** Olanzapine, Suppository, Witepsol H-15, Terminally ill patients, Delirium, Nausea and vomiting

## Abstract

**Background:**

A new formulation of olanzapine available for terminally ill patients is needed. Rectal administration using suppositories is an alternative for patients for whom administration via the oral route is not feasible. In the present study, we prepared olanzapine suppositories, and confirmed using pharmaceutical tests. Furthermore, we demonstrated the efficacy and safety of olanzapine suppositories in terminally ill patients.

**Methods:**

We prepared olanzapine suppositories using bases consisting of different compositions of Witepsol H-15, Witepsol S-55, and Witepsol E-75. The suppository release test was performed, and the olanzapine suppository with the best dissolution rate was selected. The suppository was assessed using the content uniformity test, content test in suppositories, hardness test, stability test, and clinical efficacy and safety.

**Results:**

The dissolution rate at 360 min of olanzapine suppositories with Witepsol H-15 was the best (77.0 ± 3.3 %). The suppositories prepared had a uniform weight (2.47 ± 0.02 g) and content (2.11 ± 0.07 mg). The power required to break suppositories was 7.96 ± 0.55 kgf. When olanzapine suppositories were stored with protection from light, their contents were maintained regardless of whether the temperature was at 4 °C or room temperature. The numbers of patients administered 2.5 mg, 5 mg, and 10 mg of olanzapine suppositories were 4, 19, and 1. The percentages of patients with delirium or nausea and vomiting cured with olanzapine suppositories were 82 and 57 %, respectively.

**Conclusion:**

We suggest that olanzapine suppositories prepared in the hospital by pharmacists will improve the quality of life of terminally ill patients.

**Trial registration:**

UMIN000022172. May 2, 2016 retrospectively registered.

## Background

Delirium is a common and often serious medical complication that occurs during the final 24 to 48 h of life in 85 to 90 % of terminally ill cancer patients [1, 2]. Morita et al. reported that delirium not only occurs in the final days of life, but also up to 29 days before death with a median survival time of 10 days from the onset of delirium [3]. Delirium is associated with increased morbidity in terminally ill patients, and causes distress to patients, family members, and staff [4–6]. Therefore, it is the main contributing factor to a family’s inability to continue to care for patients in their home and a common reason for admission to an inpatient hospice unit [7]. Palliative care is an approach that improves the quality of life of patients and their families facing the challenges associated with life-threatening illnesses.

Regarding pharmacological interventions, antipsychotic medication is regarded as first-line pharmacotherapy for delirium, with haloperidol, a typical antipsychotic, being the most frequently administered drug [8]. Haloperidol has fewer active metabolites and anticholinergic properties and weaker sedative and hypotensive effects than other traditional antipsychotic agents [9]. However, its use has been associated with the development of arrhythmia such as torsade de pointes and extrapyramidal effects [8, 9]. Extrapyramidal symptoms are more likely to occur in the elderly and strongly medically ill patients, who are also the most susceptible to delirium [10]. Atypical antipsychotics have a more favorable adverse effect profile due to their decreased affinity for the D_2_ dopamine receptor, which leads to weaker extrapyramidal effects [11]. Olanzapine is an atypical antipsychotic agent of the thienobenzodiazepine class. Atypical antipsychotics such as olanzapine have recently been used to treat delirious patients due to their equal effectiveness to and lower incidence of extrapyramidal symptoms than haloperidol [10, 12]. Olanzapine blocks multiple neurotransmitter receptors, including dopaminergic (D_1_, D_2_, D_3_, and D_4_), serotonergic (5-hydroxytryptamine 2A [5-HT_2A_], 5-HT_2C_, 5-HT_3_, and 5-HT_6_), adrenergic (α_1_), histaminic (H_1_), and muscarinic (M_1_, M_2_, M_3_, and M_4_) receptors [13]. The affinity of olanzapine for multiple receptors has led to its identification as an important agent in the treatment of delirium as well as nausea and vomiting [13]. Several palliative care studies have shown that olanzapine is useful and safe for the treatment of delirium [13, 14]. Sedation is commonly used to manage the symptoms of restlessness and agitation in the terminal stage of illness [15, 16]. Sedation is a common side effect in patients orally administered olanzapine, with reported incident rates of 18.9–28.6 %. Thus, olanzapine is commonly administered to terminally ill patients. However, an oral formulation is not appropriate because most patients in the final days of life cannot take drugs orally.

Previous studies demonstrated that olanzapine was not inferior to aprepitant in the prophylaxis of highly and moderately emetogenic chemotherapy and that it increased the rate of a complete response when added to a combination of a 5-HT_3_ antagonist, aprepitant, and dexamethasone [17]. However, an oral formulation is not appropriate because the ingestion of an oral drug in patients with vomiting or severe nausea may induce emesis.

Thus, a new formulation of olanzapine available for terminally ill patients is needed. Rectal administration using suppositories is an alternative for patients for whom administration via the oral route is not feasible. Rectal administration is more advantageous than intravenous administration because aseptic handling is not required and patients may administer the drugs in their own homes. Therefore, the rectal administration of an olanzapine suppository appears to be clinically useful for the treatment of terminally ill patients with delirium, vomiting, or nausea. In the present study, we prepared olanzapine suppositories using bases consisting of different compositions of Witepsol H-15, Witepsol S-55, and Witepsol E-75. The suppository release test was performed, and the olanzapine suppository with the best dissolution rate was selected. The suppository was assessed using the content uniformity test, content test in suppositories, hardness test, stability test, and clinical efficacy.

## Methods

### Materials

Zyprexa® tablets 2.5 mg (containing 2.5 mg olanzapine) were purchased from Eli Lilly Japan K. K. (Hyogo, Japan). Witepsol H-15, S-5, and E-75 suppository molds (2.25 cm^3^) were purchased from Maruishi Pharmaceutical Co., Ltd. (Osaka, Japan). Olanzapine, perchloric acid (60 %), 10 % ammonia solution, dipotassium hydrogen phosphate, and 1 mol/L potassium hydroxide solution were purchased from Wako Pure Chemical Industries, Ltd. (Osaka, Japan). Tetramethylammonium perchlorate was purchased from Tokyo Chemical Industry Co., Ltd. (Tokyo, Japan). Potassium dihydrogen phosphate and acetonitrile were purchased from Nacalai Tesque, Inc. (Kyoto, Japan).

### Preparation of the olanzapine suppository

Suppository bases consisting of different compositions of H-15, S-55, and E-75 (Table 1) were completely melted at 40 °C. Zyprexa® tablets were crushed in a mortar and sifted through a 50 mesh sieve. The crushed powder was added to the melted base and dispersed uniformly. The mixture was poured into a suppository mold and left at room temperature for 24 h to solidify.

**Table 1:** Composition of different Witepsol suppositories

Component (%)	H-15	S-55	E-75
H-15	100	-	-
H-15:S-55 = 1:1	50	50	-
H-15:S-55 = 2:1	67	33	-
H-15:S-55 = 1:2	33	67	-
S-55	-	100	-
S-55:E-75 = 1:1	-	50	50
E-75	-	-	100

### Suppository release test

The suppository release test was performed according to the reciprocating dialysis tube method with tapping using the dissolution apparatus HZ-21D (Miyamoto Riken Ind. Co., Ltd., Osaka, Japan) [18]. Phosphate buffer (pH 7.0) was used as the testing medium. The volume of the testing medium was 900 mL, and the test was performed at 37 °C. The dialysis membrane (size 27, length 21 cm) was soaked in purified water and rinsed before use. The arm of the apparatus moved up and down automatically at a constant speed (30 number/min). Four hundred microliters of the buffer was sampled at each time point (30, 60, 90, 120, 150, 180, 210, 240, 270, 300, 330, and 360 min), and 400 μL of fresh buffer was added to maintain the same volume. Samples (*n* = 6) were analyzed using high-performance liquid chromatography (HPLC).

### Measurement of olanzapine concentrations

Olanzapine concentrations were measured by HPLC with minor modifications to the methods described by Raggi et al. [19]. The analytical column was ZORBAX Eclipse Plus C8, 5 μm, 150 × 4.6 mm, the UV wavelength for olanzapine was 260 nm, and the mobile phase consisted of 17 mM tetramethylammonium perchlorate (pH 3.1):acetonitrile = 77:23. The flow rate was 0.4 mL/min.

### Content uniformity test

Ten olanzapine suppositories were selected randomly and their weights and olanzapine contents determined. A suppository was placed into a flask, 1000 mL purified water (65 °C) was added, and it was completely melted. The suspension obtained was stirred and the flask was cooled on ice to solidify the base. After cooling, the suspension was centrifuged at 3,500 rpm at 4 °C for 5 min, and the supernatant was filtered through filter paper (0.45 μm). The filtrate was analyzed by HPLC.

### Content test in suppositories

Three olanzapine suppositories were randomly selected and cut in half crosswise. The bulbous head and tail parts were obtained. Their contents were determined in the same manner as the content uniformity test.

### Hardness test

Fifteen olanzapine suppositories were randomly selected and hardness was determined using a hardness tester (EZ-test: Shimadzu Co., Kyoto, Japan).

### Stability test

Prepared olanzapine suppositories were stored under 3 conditions for 1, 2, 3, 4, 6, 8, 12, and 24 weeks: (i) at 4 °C protected from light, (ii) at room temperature (25–30 °C) protected from light, and (iii) at room temperature without protection from light. After storage, the contents of olanzapine suppositories were determined in the same manner as the content uniformity test (*n* = 3).

### Clinical application of olanzapine suppositories

This study was approved by the Ethics Review Board of Shonan Central Hospital (#0182). Written informed consent was obtained from 24 adult patients who were administered olanzapine suppositories between July 2014 and April 2015 at Shonan Central Hospital. When the administration route was changed from oral to rectal, the same dose was used. When olanzapine suppository was newly administered in terminally ill patients, a dose of 2.5 mg or 5.0 mg was determined by physicians. Responses to olanzapine therapy were evaluated based on clinical assessments by physicians. Information about the side effects such as sleepiness, mucous membrane damage caused by local irritation and diarrhea was extracted from the electronic medical records of each patient.

### Statistical analysis

Data are presented as the mean ± standard deviation (S.D.). Significance was analyzed using Dunnett’s test for multiple comparisons, and the Student’s *t*-test for two-group comparisons. Differences at *P* < 0.05 were considered significant. All statistical analyses were conducted using Microsoft Office Excel 2010 with the add-in software Statcel 3 (OMS, Inc., Hyogo, Japan).

## Results

### Suppository release test

**Fig. 1:**
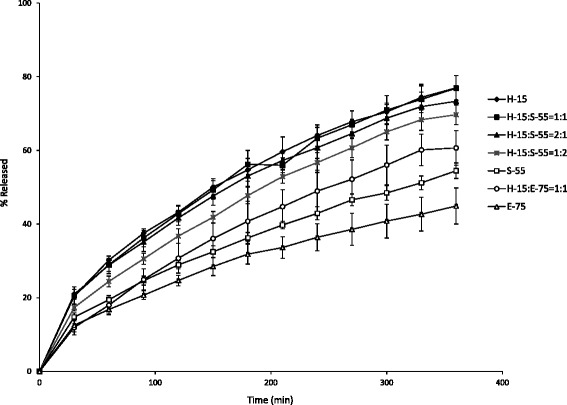
Effects of suppository bases on the release of olanzapine from suppositories. Each value represents the mean ± S.D. (*n* = 6)

The dissolution profiles of olanzapine suppositories manufactured using different compositions of H-15, S-55, and H-15 (Table 1) are shown in Fig. 1. The 50 % olanzapine release time of olanzapine suppositories with H-15, H-15:S-55 = 1:1, H-15:S-55 = 2:1, H-15:S-55 = 1:2, H-15:S-55 = 1:2, and S-55:E-75 = 1:1 as the bases were 150, 150, 165, 195, 250, and 250 min, respectively. The suppository with E-75 was not released by 360 min. The dissolution rates at 360 min of olanzapine suppositories with H-15, H-15:S-55 = 1:1, H-15:S-55 = 2:1, H-15:S-55 = 1:2, H-15:S-55 = 1:2, S-55:E-75 = 1:1, and E-75 were 77.0 ± 3.3 %, 76.9 ± 3.4 %, 73.3 ± 4.2 %, 69.7 ± 2.7 %, 54.5 ± 2.1 %, 60.7 ± 4.7 %, and 44.9 ± 4.9 %, respectively. The dissolution rates of suppositories with bases consisting of H-15 or H-15:S-55 = 1:1 were the best. Furthermore, since only H-15 was easier to prepare than the mixture of H-15 and S-55, Witepsol H-15 was selected as the base.

### Content uniformity test, content test in suppositories, and hardness test

The weight and content of olanzapine suppositories were 2.47 ± 0.02 g and 2.11 ± 0.07 mg, respectively. Content uniformity was checked using the content uniformity test laid out in the Japanese Pharmacopoeia (JP) 16th edition, and was confirmed to meet the JP criteria. The olanzapine concentrations of the bulbous head part and tail part were 1.35 ± 0.08 and 1.10 ± 0.07 mg/g, respectively. The power required to break suppositories was 7.96 ± 0.55 kgf.

### Stability test

**Fig. 2:**
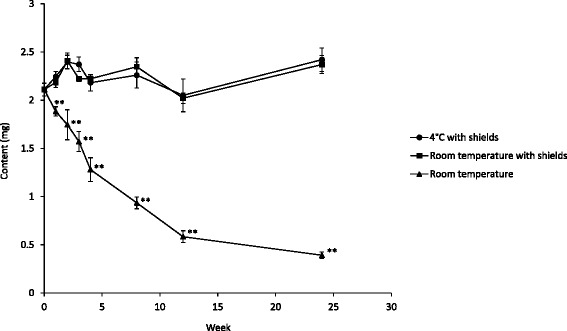
Stability of olanzapine suppositories under various storage conditions. Each value represents the mean ± S.D. (*n* = 3). **P < 0.01 significantly different from day 1 in the room temperature group

When olanzapine suppositories were stored at room temperature without protection from light, their contents significantly decreased (Fig. 2). Their contents decreased to 89 % in 1 week. In contrast, when olanzapine suppositories were stored with protection from light, their contents were maintained regardless of whether the temperature was at 4 °C or room temperature.

### Clinical effects

Patient characteristics are shown in Table 2. Fifteen men and 9 women, with a median age of 78 years, were evaluated in the present study. All patients had oral feeding difficulties. The numbers of patients changed from oral olanzapine and other oral atypical antipsychotics to olanzapine suppositories were 5 and 8, respectively. Eleven patients used olanzapine suppositories as a treatment for delirium or nausea and vomiting at the time of oral feeding difficulties. The numbers of patients administered 2.5 mg, 5 mg, and 10 mg of olanzapine suppositories were 4, 19, and 1. The median duration of the treatment was 11 days (Table 2). Table 3 shows the therapeutic efficacy of olanzapine suppositories in patients with delirium or nausea and vomiting. The percentages of patients with delirium or nausea and vomiting cured with olanzapine suppositories were 82 and 57 %, respectively. The reasons for the discontinuation of olanzapine suppositories were death (15 patients), a decreased level of consciousness (7 patients), and side effects (2 patients: sleepiness). There were no patients with mucous membrane damage caused by local irritation and diarrhea.

**Table 2:** Characteristics of 24 patients included in the present study

Characteristics	Number or median ± S.D. (range)
Sex	
Male:female	15:9
Age (years)	78 ± 10 (59–89)
Treatment duration (days)	11 ± 18 (1–73)
Underlying disease	
Lung cancer	6
Large bowel cancer	3
Pancreatic cancer	2
Bile duct cancer	2
Stomach cancer	2
Prostate cancer	2
Ovarian cancer	2
Pharyngeal cancer	1
Esophageal cancer	1
Kidney cancer	1
Bladder cancer	1
Malignant lymphoma	1

**Table 3:** Therapeutic efficacy of olanzapine suppositories in patients with delirium or nausea and vomiting

Daily dose (g)	Success	Failure	Cure rate (%)
Delirium	18	4	82
Nausea and vomiting	4	3	57

## Discussion

Suppositories need to show consistent release characteristics under the temperature range of the rectum (36.5–37.5 °C). Since olanzapine is a lipophilic drug, we selected Witepsol as the lipophilic base most widely used in clinical practice. The suppository release test was performed according to the reciprocating dialysis tube method with tapping. The dialysis tube method better simulates physiological conditions given that there is only a small amount of fluid in the rectum and showed high reproducibility and an in vitro-in vivo correlation in rabbits [18, 20]. As shown in Fig. 1, the dissolution rate at 360 min of the olanzapine suppository with H-15 was 77.0 ± 3.3 %. But, the suppository with E-75 was not released by 360 min. The melting points of H-15 and S-55 were 33.5–35.5 °C. On the other hand, the melting point of E-75 was 37–39 °C. We considered the suppository with E-75 to be difficult to dissolve at 37 °C. It keeps the degree of viscosity as low as possible in order to release the drug from the suppository [21]. Since the viscosity of H-15 was lower than that of S-55, olanzapine with H-15 was released at a more rapid rate than S-55 (Fig. 1).

The olanzapine concentrations of the bulbous head part and tail part were different in this study. Thus, dividing the suppository is not recommended. When we prepared olanzapine suppositories, the drug sometimes settled in the bulbous head part. Further studies are needed in order to achieve uniform concentrations in both parts.

A previous study reported that the power required to break original and generic acetaminophen suppositories was 5.3–8.9 kgf [22]. The hardness of the current suppositories was similar to that of other suppositories.

Although Zyprexa® tablets remained stable at room temperature when exposed to light, fine granules did not. When olanzapine suppositories were stored at room temperature without protection from light, their contents significantly decreased (Fig. 2). Therefore, olanzapine suppositories need to be stored with protection from light in order to avoid a decrease in their contents; under these conditions, their contents were confirmed to remain stable for 24 weeks.

In previous studies, 64.7–82.4 % of patients orally administered olanzapine achieved the resolution of delirium [23, 24]. In palliative care, olanzapine has been shown to control or reduce the intensity of nausea and vomiting [17]. These findings are consistent with the results of the present study (Table 3). Therefore, we herein demonstrated the efficacy of olanzapine suppositories. On the other hand, only 2 patients given suppositories discontinued due to side effects (sleepiness). Blood sampling is not routinely performed in terminally ill patients; therefore we could not check blood glucose concentrations, liver and kidney function tests, etc. with blood tests.

This study has some limitations. This was an uncontrolled study without comparative data in non-administration group. Additionally, the results of this study were obtained in a small number of patients. Therefore, further studies such as prospective randomized contolled study involving a large number of patients are needed to confirm the current findings. We were unable to analyze pharmacokinetics in patients administered olanzapine suppositories. When olanzapine was administered orally to patients at doses of 5 ± 2.5 and 10 ± 2.5 mg/day, mean blood concentrations were 10.3 ± 8.0 and 18.4 ± 12.1 ng/mL, respectively [25]. Olanzapine blood concentrations are very low. We were unable to employ HPLC with ultraviolet. Liquid chromatography tandem mass spectrometry (LC-MS/MS) is needed in order to measure olanzapine blood concentrations [26]. Another reason is the lower frequency of blood sampling in terminally ill patients with cancer. Therefore, we were unable to obtain blood concentrations. Previous studies reported that the mechanism underlying drug absorption from the rectum may not differ from that in the upper part of the gastrointestinal tract [27, 28]. The hepatic first-pass elimination of high clearance drugs is partly avoided with rectal administration. After oral administration, 85 % of olanzapine is readily absorbed; however, due to high first-pass hepatic metabolism, its oral bioavailability is only 60 % [29]. Thus, patients given suppositories may have higher blood concentrations than those receiving drugs via an oral route.

We considered that suppositories would be able to be prepared more easily by the use of solutions of risperidone. Furthermore, the clinical response rates to delirium did not differ significantly between olanzapine and risperidone [23, 24]. But, olanzapine-induced sedation occurred more frequently than risperidone [23]. So, we selected olanzapine, because sedation is commonly used to manage the symptoms of restlessness and agitation in the terminal stage of illness [15, 16].

## Conclusion

We prepared olanzapine suppositories, and demonstrated the efficacy in terminally ill patients. Rectal administration using suppositories is an alternative for patients for whom administration via the oral route is not feasible. We suggest that olanzapine suppositories prepared in the hospital by pharmacists will improve the quality of life of terminally ill patients. But, we were unable to analyze pharmacokinetics in patients administered olanzapine suppositories. Although the number of blood sampling should be the minimum for terminally ill patients, olanzapine blood concentrations will need to be measured with LC-MS/MS to assure the safety in the future.

## Abbreviations

E-75, Witepsol E-75; H-15, Witepsol H-15; HPLC, high-performance liquid chromatography; LC-MS/MS, liquid chromatography tandem mass spectrometry; S-55, Witepsol S-55
